# Health care centre and emergency department utilization by patients with episodes of tachycardia

**DOI:** 10.1186/s12872-022-02568-y

**Published:** 2022-03-23

**Authors:** Ann-Katrin Nordblom, Gabriella Norberg Boysen, Mia Berglund, Anna Kjellsdotter

**Affiliations:** 1 Department of Cardiology, Skaraborg Hospital Skövde, 54185 Skövde, Sweden; 2 Faculty of Caring Sciences, Work Life and Social Welfare, University of Borås, Borås, Sweden; 3 PreHospen - Centre for Prehospital Research, University of Borås, Borås, Sweden; 4 School of Health Sciences, Skövde University, Skövde, Sweden; 5 Research and Development Centre, Skaraborg Hospital Skövde, Skövde, Sweden

**Keywords:** Episodes of tachycardia, Arrhythmia, Health care centre, Emergency department, Sex and age differences, Retrospective study

## Abstract

**Background:**

Outpatients seek to visit health care facilities for episodes of tachycardia-related signs and symptoms. The challenge for physicians is to balance a proper initial assessment and avoid overlooking a possible arrhythmia. This common clinical situation affects individuals and health care utilization, and effective management may substantially affect health care resources. This study aimed to explore health care utilization for outpatients with episodes of tachycardia visiting health care centres (HCCs) and/or emergency departments (EDs).

**Method:**

This retrospective study used data of adult outpatients (≥ 18 years) who were assessed by a physician as having a specific or nonspecific diagnosis of arrhythmia between 2017 and 2018, and data were retrieved from medical records and a regional registry database. Data was analysed with appropriate statistical analyses to identify disparities between sex, age and terms of search pattern for each health care facility. Analysis of variance was used to test disparities between the sexes, and one-factor ANOVA was used for the incidence of missed arrhythmias.

**Results:**

A total of 2719 visits with 2373 outpatients were included in the study. The result showed a significant difference in the total number of visits (*n* = 2719) between female and male patients (68% vs. 32%, *p* < .001). In the 60–69- and 70–79-year age groups, females had significantly higher frequencies of visits than males (*p* = .018). A significant difference was also observed between sexes in terms of which health care facility they tended to visit (*p* < .001). Ninety-five percent of the outpatients visiting EDs were hospitalized. When estimating the incidence of missed arrhythmias (diagnoses) in relation to assessments, the results showed a 5% missed diagnosis involving potential atrioventricular nodal re-entry tachycardia and atrioventricular re-entry tachycardia. Moreover, the referral rate was low, especially from HCCs to cardiologists.

**Conclusions:**

This study shows a significant difference in total visits in HCCs and/or EDs by patients of different sexes and indicates the need for improved care for outpatients with episodes of tachycardia. Sex- and age-related differences must be addressed with an aim of providing equal care. Finally, the low rate of referral from HCCs to cardiologists compared to the high proportion of hospitalizations from EDs, deserves further investigation.

## Background

Episodes of rapid heart rate with tachycardia-related signs and symptoms are a reason why people seek medical attention at health care centres (HCCs) or emergency departments (EDs) [1–3]. The episodes may affect patients’ daily lives, as well as their need for medical assistance, generating consequences for individuals (repeated investigations/visits, social disadvantages, and economic costs) [4–6] and society (costs to the national economy and the consumption of health care resources) [5, 7, 8]. The severity of the episodes and the presence of signs and symptoms is highly variable, depending on the patient’s heart rate, duration of tachycardia, underlying heart disease, and individual experience [1, 9]. Supraventricular tachycardia (SVT) is a generic term for atrial arrhythmias, including condition such as atrioventricular nodal re-entry tachycardia (AVNRT) and atrioventricular re-entry tachycardia (AVRT). SVT affects approximately 0.17–0.20% of the Western population [10–12]. These arrhythmias are benign, except in rare situations [1, 9, 13]. They may occur at any age; although they are common in young adults, they do not emerge in some patients until they are elderly. Females are diagnosed with AVNRT more frequently than males (70% vs. 30%); the opposite is true for AVRT (45% vs. 55%) [1, 14, 15]. The signs and symptom are often recognizable for the patient from previous episodes and may be associated with palpitations, neck pounding, light-headedness, chest discomfort and presyncope, and rarely syncope [1, 3]. Symptoms, such as fatigue and/or polyuria, may occur during or after an episode of tachycardia and may last for hours or even days [4]. The clinical characteristics of AVNRT and AVRT are the abrupt onset and sudden termination of regular tachycardia [1, 3]. Episodes may be more frequent with longer durations over time and may exert a severe, negative effect on a patient’s quality of life with an increased symptom burden [1, 9]. Previous research has shown a negative effect on patients’ daily lives [6, 14–16] and patient frustration due to repeated, time-consuming visits to health care services and increased utility of health care resources [3, 6, 14]. Patients have experienced not being taken seriously by physicians, with their signs and symptoms explained as stress or mental instability [4, 6, 14, 15]. The challenge for physicians is to balance a proper initial assessment and estimation of the likelihood of a relevant underlying arrhythmia or other possible explanation [1–3]. Electrocardiograms (ECGs) with ongoing SVT show a characteristic tachycardia pattern, while this finding is valuable for the assessment, it can be challenging since the episode may be terminated spontaneously before 12-lead ECG is recorded [4, 15]. Overlooking a possible arrhythmia may cause a delay in proper first-line treatment for AVNRT and AVRT, for example, vagal manoeuvres as self-management [1, 3, 5, 17, 18] and a delay in referral for a cardiologist opinion [14, 15]. Patients need information about how their symptoms and quality of life may change following these treatments to enable them to make informed decisions about treatment options [1, 18, 19]. Catheter ablation therapy represents a potentially curative therapy, with a 95–97% success rate for AVNRT and a 95% rate for AVRT [1, 14, 18], compared to lifelong drug treatment [1, 18]. Following catheter ablation, a significant improvement is observed in a variety of symptoms and quality of life indices [10, 13, 19], as well as decreases in medical and health care costs [20, 21]. However, studies of health care utilization at both HCCs and EDs for outpatients with episodes of tachycardia (potential SVT) are limited [5, 15]. Hence, effective management of outpatients with tachycardia may substantially affect the utilization of health care resources [2, 21]. This study aimed to explore health care utilization by outpatients with episodes of tachycardia visiting HCCs and/or EDs. The hypothesis is that no differences in search patterns exist between sexes at health care facilities.

## Method

Sweden has a long tradition of health data registration based on the personal identity number that each citizen is assigned at birth or when attaining citizenship. This retrospective registry study was conducted in southwestern Sweden with an approximate population of 214 000 inhabitants aged ≥ 18 years (50.5% male) [22]. This area has two county hospitals with EDs and 15 communities, each with one or several HCCs [22]. The HCC constitutes the first level of care; it is considered an entry point for patients with new health problems and, if necessary, collaborates with other levels of care and specialists to coordinate patient care and treatment [23]. Since 1992, the International Classification of Diseases and Related Health Problems (ICD-10) [24] has been used to statistically classify groups of diseases, health problems and causes of death in medical records. The 10th Revision Swedish version (ICD-10-SE) [24] was used in this study. Permission was obtained by the Ethical Review Board of Uppsala, Sweden (Dnr: 2019–03,295 with additional application 21-01-2020). The permit includes that need for written consent was waived, due to the retrospective nature of the study. The investigation conforms with the principles outlined in the Declaration of Helsinki [25].

### Data collection

Data were collected using statistical data for outpatients´ unplanned visits to HCCs and EDs during the period from January 2017 to December 2018. Data were selected from a registry (regional administrative health care database) and medical records. Initially, the outpatients were identified by their identification number, and then participant data were anonymised, but these changes did not distort the scientific meaning. The inclusion criteria were outpatients aged ≥ 18 years who were diagnosed by a physician as having a specific or nonspecific arrhythmia that included at least one of the selected ICD codes described in the next section. The exclusion criteria were a known diagnosis of atrial fibrillation or atrial flutter (ICD-10-SE: I480, I481, I482, I489) [24].

### Selection of ICD-10-SE codes

Selected ICD-10-SE codes were used to identify potential outpatients with symptoms and signs related to episodes of tachycardia (potential SVT) [24]. The sample comprised R-codes of nonspecific symptoms related to tachycardia and the I-codes with nonspecific or specific diagnoses related to SVT, as follows: I471, ‘Paroxysmal supraventricular tachycardia’; I479, ‘Paroxysmal tachycardia, unspecific’; I498, ‘Other specific heart-arrhythmia’; and I499, ‘Heart-arrhythmia, unspecific’. The nonspecific symptom R-codes were identified as common in the medical record for the patient group of interest and considered important to identify patients without a diagnosis of arrhythmia, as follows: R000, ‘Tachycardia, unspecific’ and R002, ‘Palpitations’. Among the outpatients visiting EDs because of arrhythmia, another ICD-10-SE code of interest was identified—U999, ‘Diagnostic information is missing’- because of the high incidence of measures related to the heart and resulting of hospitalization for further investigation.

### Validation of heart-rhythm

Random sampling was conducted using 100 outpatients admitted to EDs with unspecific ICD-10-SE codes (R000, R002 and U999) to estimate the incidence of missed arrhythmias (diagnoses) in relation to the ECG assessment. The accuracy of the sample was determined based on 95% in confidence interval (CIs). It was distributed as a percentage by age group and sex using a random generator in the Excel software program (Random Generator at Microsoft Excel, Office 365). The intention was to identify arrhythmia as AVNRT or AVRT (ICD-10-SE codes: I45.6A and I47.1, respectively) [24]. The inclusion criteria were adherence to the criteria and confirmation by the electrophysiologist. A review of the records was performed for all potential cases using an algorithm based on standard ECG criteria for SVT to interpret ECG and then identify the potentially eligible arrhythmia. The criteria were as follows: (1) paroxysmal occurrence (sinus and episodes of tachycardia), (2) narrow QRS complex configuration or pre-excited bundle branch block, (3) variation in successive RR ≤ 40 ms, (4) ventricular rate ≥ 120 bpm, (5) no evidence of AV dissociation and (6) no identifiable P-waves preceding the QRS complex during tachycardia [11]. The ECG identified as potentially eligible AVNRT or AVRT was then confirmed by the electrophysiologist.

### Data analysis

Descriptive statistics were used to assess the participants’ visits after stratification by sex, age, frequency, level of care (HCC and ED) by calculating mean values and 95% CIs. The difference between sexes was pronounced even in the 10-year age groups. Analysis of variance (ANOVA) was used to determine the statistical significance of differences. We included one factor, incidence of arrhythmias, as the number of unique patients per 10 000 inhabitants, and one-factor ANOVA was used. The non-parametric Mann–Whitney U test was used for the variables that were not normally distributed. The chi-square test was used to compare categorical variables and identify disparities between sex, age and terms of the search pattern for each health care facility, as well as the proportions. A *p* value of ≤ 0.05 was considered statistically significant. Statistical analysis was performed using SPSS/WIN v24.0 software.

## Results

**Fig. 1:**
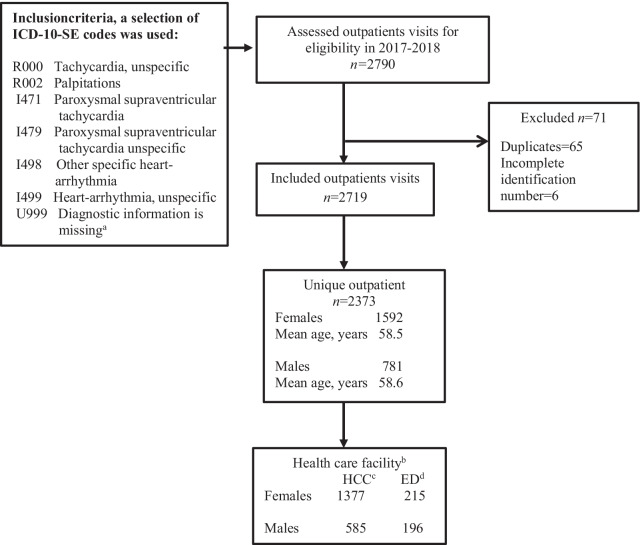
Flowchart of the study process. ^a^Selected ICD-10-SE code motivated of the high incidence of measures related to the heart rhythm, ^b^first index visit, ^c^health care centre, and ^d^emergency department

This study was conducted in a geographical area with inhabitants ages ≥ 18 years (male 50.5%, female 49.5%) and was designed to explore the current situation for adult outpatients with episodes of tachycardia visiting HCCs and/or EDs (Fig. 1).

**Fig. 2:**
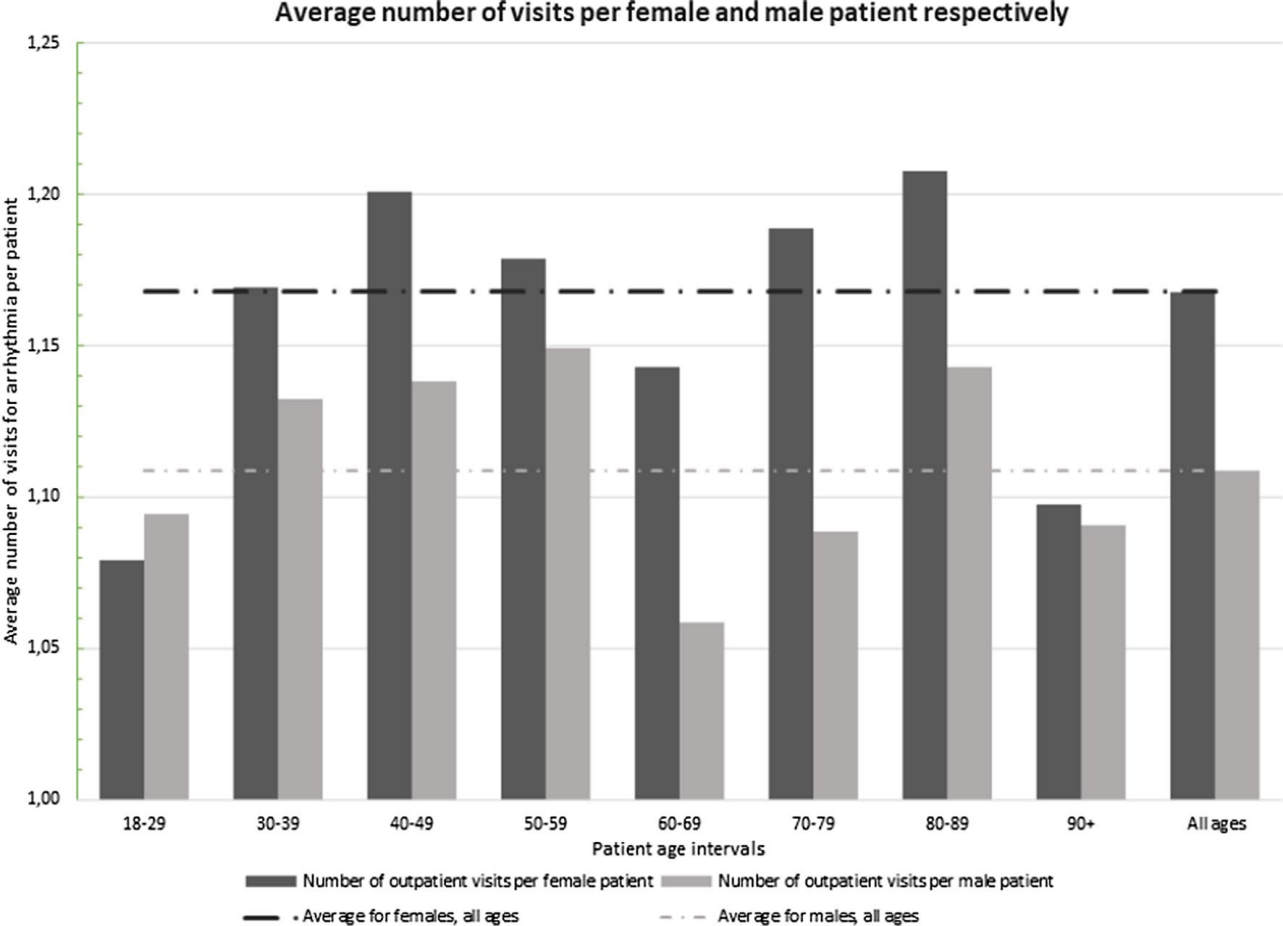
Outpatient visits (*n* = 2719) to health care centres and/or emergency department due to episodes of tachycardia in groups stratified by sex and age (18–99 years)

In total, 2790 visits were eligible for the study; of these, 65 were duplicates and six were excluded due to an incomplete identification number. Hence, 2719 visits were included, consisting of 2373 outpatients, of which 1592 (67%) were female and 781 (33%) were male. Of the 2719 outpatient visits, 82% were at HCCs, and 18% were at EDs. The differences between sexes were pronounced when the sample of outpatients was divided into 10-year age groups. By extracting demographic data for sex and age groups from Statistics Sweden for 2017 [22], we were able to calculate the number of patients per 10 000 inhabitants in each age group. At the aggregate level, 112 out of 10 000 inhabitants had at least one visit to an HCC or ED because of episodes of tachycardia in 2017–2018; thus, 1.12% of the inhabitants visited a provider at least once. The results showed a significant difference in the total number of visits (*n* = 2719) between female and male outpatients (68% vs. 32%, *p* < 0.001). A significant difference was observed between sexes; among the females, 151 of 10 000 had visited at least once because of the condition, whereas the corresponding number among males was 73 (*p* < 0.01). When analysed by the average and unique persons, female outpatients had 1.17 visits, and male outpatients had 1.11 visits. In the 60–69- and 70–79-year age groups, females had significantly higher frequencies of visits than males (*p* = 0.018) (Fig. 2).

A significant difference (*n* = 2719) in the type of health care facility outpatients visited was observed between the sexes (*p* < 0.001). A significant difference between the unique outpatient (*n* = 2373) female and male visits to HCCs and visits to EDs was also observed (*p* < 0.001). Further, a significant difference between sexes was observed among the age groups who visited HCCs (*p* < 0.001). However, more females than males in the ≥ 66-year age group (*p* < 0.001) sought care at EDs, but the opposite result was obtained for the 18- to 65-year age group. In general, the outpatients who sought care at EDs were considerably older than those seeking care at HCCs (Table 1).

**Table 1:** Outpatient visits to HCCs^a^ and/or EDs^b^ stratified by sex and age

	Total	Visits to HCCs	Visits to EDs	*P* value
*Visits*	*n* = *2719*	*n* = *2239*	*n* = *480*	
Female (%)	1857 (68)	1596 (71)	261 (54)	< .001^d^
Male (%)	862 (32)	643 (29)	219 (46)	
*Unique outpatients*^c^	*n* = *2373*	*n* = *1962*	*n* = *411*	
Female (%)	1592 (67)	1377 (70)	215 (52)	< .001^d^
Male (%)	781 (33)	585 (30)	196 (48)	
Mean age of females, years (SD)	58.5 (18.9)	56.0 (18.1)	73.3 (16.6)	< .001^e^
Mean age of males, years (SD)	58.6 (18.4)	54.3 (17.9)	70.6 (13.7)	< .001^e^
Age 18–65 years	*n* = *1383*	*n* = *1283*	*n* = *100*	
Female (%)	931 (67)	891 (69)	40 (40)	< .001^d^
Male (%)	452 (33)	392 (31)	60 (60)	
Age ≥ 66 years	*n* = *990*	*n* = *679*	*n* = *311*	
Female (%)	661 (67)	486 (72)	175 (56)	< .001^d^
Male (%)	329 (33)	193 (28)	136 (44)	

^a^Health care centre

^b^Emergency department

^c^First index visit

^d^The chi-square test was conducted to compare categorical variables between groups

^e^Mann–Whitney test was employed to compare continuous data between groups

A range of 1–9 visits per person was recorded for the 2373 outpatients. A comparison of visits to health care facilities resulted in a number of 1.12 visits to HCCs and 1.06 visits to EDs. The most frequently used ICD-code was R002 (Palpitations), which was assessed in 1759 visits from 1569 outpatients, with a quota of 1.12 (Table 2).

**Table 2:** The ICD-10-SE^a^ codes used as diagnostic description at HCCs^b^ and EDs^c^

ICD-10-SE code	Diagnostic description	Visits to HCCs *n* (%)	Visits to EDs *n* (%)	Patients treated at HCCs *n*	Patients treated at EDs *n*	Number of visits per patient
I471	Paroxysmal supraventricular tachycardia	55 (2)	8 (2)	50	7	1.11
I479	Paroxysmal tachycardia, unspecific	43 (2)	6 (1)	40	6	1.07
I498	Other specific heart-arrhythmia	49 (2)	6 (1)	46	6	1.06
I499	Heart-arrhythmia, unspecific	60 (3)	68 (14)	50	68	1.08
R000	Tachycardia, unspecific	309 (14)	15 (3)	287	15	1.07
R002	Palpitations	1723 (77)	36 (8)	1534	35	1.12
U999^d^	Diagnostic information is missing	0 (0)	341 (71)	0	316	1.08
Total		2239	480	2007	453	1.11
Number of visits per unique patient		1.12	1.06			

^a^International Classification of Diseases and Related Health Problems, 10th Revision, Swedish version

^b^Health care centres

^c^Emergency departments

^d^U999 was only reported in the documentation for EDs

A total of 277 outpatients with episodes of tachycardia had 623 recurrent visits at HCCs and/or EDs. These outpatients had ≥ 2 visits in under 24 months, with a range 2–9 visits per outpatient; the visits were more common among females (*p* = 0.001). This result shows a higher frequency of visits to HCCs (487) than to EDs (56). Moreover, 35 outpatients (20 females and 15 males) visited both HCCs and EDs, with a total of 80 visits and a mean number of 2.3 visits (Table 3).

**Table 3:** Recurrent visits to HCCs^a^ and/or EDs^b^ by outpatients stratified by sex and age

Recurrent visits, *n* = number of visits (%)	Total *n* = *623*	Visits to HCCs *n* = 487	Visits to EDs *n* = 56	Visits to both HCCs and EDs *n* = 80
*Unique persons (%)*	*277*	218 (78.7)	24 (8.7)	35 (12.6)
Age 18–65 years	*n* = 162	*n* = 143	*n* = 4	*n* = 15
Female (%)	118 (73)	107 (75)	2 (50)	9 (60)
Male (%)	44 (27)	36 (25)	2 (50)	6 (40)
Age ≥ 66 years	*n* = 115	*n* = 75	*n* = 20	*n* = 20
Female (%)	92 (80)	65 (87)	16 (80)	11 (55)
Male (%)	23 (20)	10 (13)	4 (20)	9 (45)
Number of visits per person (range)	2.2 (2–9)	2.2 (2–9)	2.3 (2–4)	2.3 (2–5)

Recurrent visits = an outpatient with ≥ 2 visits due to recurring tachycardia

^a^Health care centres

^b^Emergency departments

Four hundred fifty-four outpatients (248 females and 206 males) were hospitalized among 480 visits, corresponding to 95% of the population recurrently visiting EDs due to episodes of tachycardia. No significant difference in hospitalization was observed between the sexes, either in the whole sample or after stratification by age group. For outpatients admitted from EDs (*n* = 454), the ICD code U999 was most common diagnosis (*n* = 336), followed by I499 (*n* = 61) (Table 4).

**Table 4:** Hospitalization for outpatients visiting EDs^a^, stratified by sex and age

	Hospitalization No	Hospitalization Yes	Total group
Visits	*n* = *26*	*n* = 454	*n* = 480
Female (%)	13 (50)	248 (55)	261 (54)
Male (%)	13 (50)	206 (45)	219 (46)
Age 18–65 years	*n* = 18	*n* = 103	*n* = 121
Female (%)	10 (56)	42 (41)	52 (43)
Male (%)	8 (44)	61 (59)	69 (57)
Age ≥ 66 years	*n* = 8	*n* = 351	*n* = 359
Female (%)	3 (38)	206 (59)	209 (58)
Male (%)	5 (62)	145 (41)	150 (42)

^a^Emergency departments

Three outpatients visiting HCCs were referred to a cardiologist; referral for 289 outpatients occurred internally at the hospital. Regarding the total numbers of referrals for outpatients, 166 were female and 126 were male, with the highest proportion of referrals occurring in the 60–69-year age group (44 females, 33 males).

Of the 2719 visits to HCCs and EDs, 2424 visits were classified with the nonspecific ICD-10-SE symptom codes R000, R002 and U999 (Table 2). In the group visiting EDs, 392 visits consisting of 366 unique outpatients were identified (Table 5). Due to limited access to data from HCC, a random sample was collected from the group of outpatients’ records admitted to EDs. This random sample consisted of ECG from a total of 100 unique outpatients distributed into four groups based on sex and age. The analysis of ECGs identified 5% (CI 1.9%-10.6%) of arrhythmias as potential AVNRT or AVRT, of which 4% occurred in females. The remaining ECG analyses were atrial fibrillation/atrial flutter (56%), sinus-rhythm (26%) and other arrhythmias (13%) (Table 5).

**Table 5:** Documentation for outpatient at ED^a^ with unspecific codes R000, R002 and U999

Patient	Age 18–65 years *n* = 92 (%)	Age ≥ 66 years *n* = 274 (%)	Total *n* = 366 (%)
Female	38 (41)	155 (57)	192 (53)
Male	54 (59)	119 (43)	172 (47)
ECG-rhythm	*n* = 28 (%)	*n* = 72 (%)	*n* = 100 (%)
AVNRT/AVRT	4 (14)	1 (1)	5 (5)
Atrial fibrillation/flutter	15 (54)	41 (57)	56 (56)
Other arrhythmia	2 (7)	11 (15)	13 (13)
Sinus	7 (25)	19 (27)	26 (26)

^a^Emergency departments

## Discussion

The result from the present study showed a significant difference in the total number of visits (*n* = 2719) between females and males (67% vs. 33%, *p* < 0.001). In the analysis of unique outpatients (*n* = 2373), significant differences were observed in the numbers of visits to HCCs (70% vs. 30%, *p* < 0.001) and visits to EDs (52% vs. 48%, *p* < 0.001) between females and males. Our hypothesis is that no difference in search patterns exist between sexes at health care facilities. The result showed a significant difference in the health care facility the outpatient tended to visit between sexes (*p* = 0.001); therefore, the hypothesis was rejected. Moreover, 82% of the 2719 visits were at HCCs, and 18% were at EDs. Thus, at an aggregate level, 1.12% (112 of 10,000 inhabitants) had visited an HCC or ED at least once over a period of 24 months. The average number of visits for females of all ages was 1.17; it was 1.11 for males. A clear difference was observed between sexes; of the 10,000 inhabitants, 151 females and 73 males had visited a health care facility at least once because of the condition (*p* < 0.01). The result can be interpreted in relation to earlier research showing that the incidence of SVT is slightly higher in females than in males [11, 12, 21]. Furthermore, health care utilization in HCCs [21, 26] and EDs [27] is more common among females. The total per capita cost for health care is higher for females, with a cost difference of 8% after adjusting for reproduction and sex-specific morbidity [26].

As shown in the present study, more than one visit (range of 2–9) in less than 24 months was recorded for 277 outpatients in 623 visits, which were mainly females visiting HCCs. Furthermore, 35 outpatients made 80 visits in both HCCs and EDs (20 females, 15 males). Previous research has reported the consequences for both patients and society due to repeated visits to a health care facility [8]. Effective management of SVT, such as early identification and response to symptoms, may substantially affect health care utilization for this group of patients [2, 21].

The results showed that 95% of outpatients visiting EDs were hospitalized, and no significant differences were observed for groups stratified according to sex or age, although hospitalization was more common among outpatients aged ≥ 66 years. A recently published study reported that SVT is associated with significant higher annuals rates of ED visits, physician office visits, hospitalization, and diagnostic testing [5]. According to previous studies, the hospitalization rate increased with advancing age, with the highest admissions reported in patients aged 80–84 years and a slightly higher presence in females [12, 13]. As the intention of health care is to provide available and equal access to care [23], this group is an important population on which to focus.

This study estimated the incidence of missed arrhythmias (diagnoses) in relation to assessment in a random sample of 100 outpatients admitted to the ED. The results showed a 5% missed diagnosis involving potential AVNRT or AVRT, which is comparable to the findings reported in the study by Orejarena et al. [11]. Thus, diagnoses for outpatients may be delayed by the clinical assessment [4, 14]. The current study did not consider the individual physician’s ability to interpret arrhythmias in an ECG; the ECG may have been read by a junior doctor or a specialist. Furthermore, the result of 5% missed arrhythmias (diagnosis) occurring in four females and one man might be due to random selection. Regardless, the results should be considered based on previous research reporting that females are likelier to receive more accessible, less expensive primary care, while males were more likely to receive specialist inpatient care [26], as well as other data showing a significantly longer history of symptomatic SVT for females before catheter ablation than for men [12]. Recently published studies concluded that SVT imposes a substantial economic burden on health care systems [4, 28] and is consistent with the burden attributed to atrial fibrillation [28, 29].

The results of the current study show a higher incidence of nonspecific ICD codes, especially symptom code R002. Previous research has shown the potential utility of selected diagnostic codes in inpatient and ED settings for identifying symptomatic SVT [10]. For patients visiting health care facilities, despite their own insecurity about the meaning about the symptoms [6, 15], they unfortunately encounter health care providers who attribute their palpitation and related symptoms to underlying causes other than SVT [14, 27]. Previous studies have described the difficulties these patients encounter when trying to prove their symptoms before their arrhythmia is confirmed on an ECG [4, 6, 14]. Obtaining a diagnosis seems important because of the need to treat these patient’s and conceivably refer them to a cardiologist [1, 9, 14].

The present study found a low referral rate; only three outpatients were referred to a cardiologist from the HCC. Of the 292 referrals, 57% were female outpatients, suggesting that the number of referrals is equal; however, given the large percentage of women who presented with the condition, the referral rate should be considered. The low referral rate is consistent with previous studies [14, 15]. The consequences of a low referral were presented as unnecessary investigation and admissions for patients with SVT presenting to the ED [30] compared to the current study, where patients mainly presented to HCCs. Sex-related differences are significant in terms of SVT diagnosis and management [14, 21, 27]. Overall, females had to wait for 60 months for catheter ablation after the onset of initial symptoms compared with 15 months for males [27]. Current knowledge is lacking regarding the delay in referral for an indicated, first-line treatment procedure such as catheter ablation [27]. In previous studies, a previous visit to an electrophysiologist and following catheter ablation significantly reduced health care utilization [21] among patients presenting to the ED with SVT [2].

The results provide a glimpse of the health care resource and group levels burdens, but the personal burden due to repeated visits to healthcare facilities for episodes of tachycardia is not difficult to imagine. Patients’ subjective experiences have been described in previous research [4, 6, 15]; however, they require further in-depth consideration, and upcoming studies are planned.

## Limitations

Although our study contributes to knowledge about disparities in adult outpatients with episodes of tachycardia stratified by sex and ages, it has several limitations. First, this study was based on retrospective data obtained from medical records and registers to explore health care utilization for a period of 24 months. Clinical presentation of episodes of tachycardia was based on selected ICD code assessed by the physician. The categorical use of ICD code requires further investigation for evaluate their validity. Second, the population consisted of outpatients visiting HCC and ED facilities that did not appear to have been explored at the time, except for a few identified studies [5, 21, 28]. Due to the availability of data, the subgroup analysis was conducted only with ECGs from outpatients admitted to ED, which may be a limitation. The results for the health care utilization of outpatients with episodes of tachycardia to determine the health care burden requires further confirmation by examining patient-centred outcomes and cost-effectiveness data. Another limitation, referrals, may have reached the cardiologists through other routes that are unknown to the authors of present study. Finally, as with all studies, the generalizability of the results to a similar context and to other countries must be valued and assessed by the reader. This study was performed in a Swedish context, which might be a limitation.

## Conclusion

As shown in the present study, females had a higher health care utilization rate than males, especially in the ≥ 66-year age group, and mainly in HCCs. The sex- and age-related differences provide an interesting body of evidence for clinical practice and future research. The result of this study is noteworthy due to clinical workup and illuminate the need for development of an early assessment of potential recurrent arrhythmias. Finally, the low referral rate to specialist care from HCCs compared to the high proportion of patients’ hospitalization from EDs deserves further investigation.

## Data Availability

The dataset generated and/or analysed during the current study are not publicly available but available from the corresponding author on reasonable request.

## Abbreviations

ANOVA: Analysis of variance
AVNRT: Atrioventricular nodal re-entry tachycardia
AVRT: Atrioventricular re-entry tachycardia
AV: Atrioventricular
CI: Confidence interval
ECG: Electrocardiogram
ED: Emergency department
ICD-10-SE: International Classification of Diseases and Related Health Problems, the 10th Revision Swedish version
HCC: Health care centre
SVT: Supraventricular tachycardia
RR: Specific part of a heartbeat, the R-wave (measuring R-wave to R-wave)

## Declarations

### Content to participate

The need for written consent was waived by the Ethical Review Board of Uppsala, Sweden, due to the retrospective nature of the study. The Ethical Review Board of Uppsala, Sweden have approved the registry protocols in this study.
